# Hydrogen Nanobubble Water Delays Petal Senescence and Prolongs the Vase Life of Cut Carnation (*Dianthus caryophyllus* L.) Flowers

**DOI:** 10.3390/plants10081662

**Published:** 2021-08-12

**Authors:** Longna Li, Qianlan Yin, Tong Zhang, Pengfei Cheng, Sheng Xu, Wenbiao Shen

**Affiliations:** 1 Laboratory Center of Life Sciences, College of Life Sciences, Nanjing Agricultural University, Nanjing 210095, China; lln2013034@njau.edu.cn (L.L.); 10117118@njau.edu.cn (Q.Y.); 2020116098@stu.njau.edu.cn (T.Z.); 2020216037@njau.edu.cn (P.C.); 2 Institute of Botany, Jiangsu Province and Chinese Academy of Sciences, Nanjing 210014, China; xusheng@cnbg.net; 3 Center of Hydrogen Science, Shanghai Jiao Tong University, Shanghai 200240, China

**Keywords:** hydrogen nanobubble water, vase life, senescence-associated enzymes, cut carnation flowers

## Abstract

The short vase life of cut flowers limits their commercial value. To ameliorate this practical problem, this study investigated the effect of hydrogen nanobubble water (HNW) on delaying senescence of cut carnation flowers (*Dianthus* *caryophyllus* L.). It was observed that HNW had properties of higher concentration and residence time for the dissolved hydrogen gas in comparison with conventional hydrogen-rich water (HRW). Meanwhile, application of 5% HNW significantly prolonged the vase life of cut carnation flowers compared with distilled water, other doses of HNW (including 1%, 10%, and 50%), and 10% HRW, which corresponded with the alleviation of fresh weight and water content loss, increased electrolyte leakage, oxidative damage, and cell death in petals. Further study showed that the increasing trend with respect to the activities of nucleases (including DNase and RNase) and protease during vase life period was inhibited by 5% HNW. The results indicated that HNW delayed petal senescence of cut carnation flowers through reducing reactive oxygen species accumulation and initial activities of senescence-associated enzymes. These findings may provide a basic framework for the application of HNW for postharvest preservation of agricultural products.

## 1. Introduction

The rapid senescence of cut flowers during postharvest periods limits their economic value. Flower senescence is a coordinated and complex process, which is primarily related to loss of water, leakage of ions, generation of reactive oxygen species (ROS), and synthesis and degradation of proteins and nucleic acids [1]. ROS are involved in membrane degradation and contribute to the cell death. During the postharvest period for cut flowers, ROS overproduction was commonly observed, while scavenging of ROS may delay the onset of cut flower senescence as a result of increasing activities of antioxidant enzymes [1,2,3].

In the petals of *Petunia* flowers, activities of five nucleases increased [4], and RNase activity in petals of *Hemerocallis* did so during senescence [5]. Additionally, the degradation of protein exhibits a crucial role in the flower senescence, commonly accompanied with increased protease activity [1]. Accordingly, chemical inhibition of protease delayed the time to visible senescence in *Sandersonia* [6] and *Iris* [7] flowers. 

Hydrogen gas (H_2_), considered to be a selective antioxidant, has so far been primarily used in medicine [8,9]. Interestingly, the production of H_2_ has been observed in plants under the normal or stressed conditions [10,11,12], although its detailed synthetic pathway (s) are not fully elucidated. Further studies have revealed that H_2_ played an important role in defense responses of plants to abiotic stresses [13,14,15], plant growth [16], and secondary metabolism [17]. H_2_ was beneficial to postharvest preservation of fruits (kiwifruit [18,19] and tomato [20]) and cut flowers (rose [21], lily [22], and *Lisianthus* [23]). Previous studies have reported that H_2_, dissolved in water [24] or supplied by a H_2_-releasing material (magnesium hydride [MgH_2_]) [25,26], can prolong the vase life of cut carnation (*Dianthus Caryophyllus* L.) flowers by enhancing activities of antioxidant enzymes and by the involvement of other gaseous signaling molecules (including nitric oxide and hydrogen sulfide).

At present, hydrogen-rich water (HRW) is a main source for exogenous H_2_ delivery, while H_2_ originates mainly from electrolytic water or gas cylinder [27]. However, the low solubility and short residence time of dissolved H_2_ limits its wide-spread application. Fortunately, the nanobubble technology provides an opportunity to overcome these disadvantages. Nanobubbles (less than 500 nm in diameter) have several properties including large surface area, high internal pressure, and negatively charged surface (zeta potential), which accelerate dissolution of the gas into the liquids and remain its stability in the liquids for longer times [28]. Hydrogen nanobubble water (HNW) was observed to exhibit higher antioxidant activity than conventional HRW without nanobubbles [29]. Previous results showed that HNW improved copper tolerance in *Daphnia magna* by reducing oxidative damage [30]. Although solid H_2_-storage materials (such as MgH_2_) can also improve the solubility and residence time of H_2_ in water, their potential threat to the environment should be concerned, especially when they are extensively used in agriculture. Thus, without conventional chemical additives except H_2_, HNW is more environmentally friendly. 

In this study, we aimed to identify the benefits of HNW in prolonging the vase life of cut carnation flowers. It was confirmed that HNW was a superior source to delay cut flower senescence in comparison with HRW. Further experiments showed that HNW reduced ROS accumulation and the initial activities of DNase, RNase, and protease. These findings provide a basic idea for application of HNW in postharvest preservation of agricultural products.

## 2. Results

### 2.1. Effects of HNW on the Vase Life of Cut Carnation Flowers

**Figure 1:**
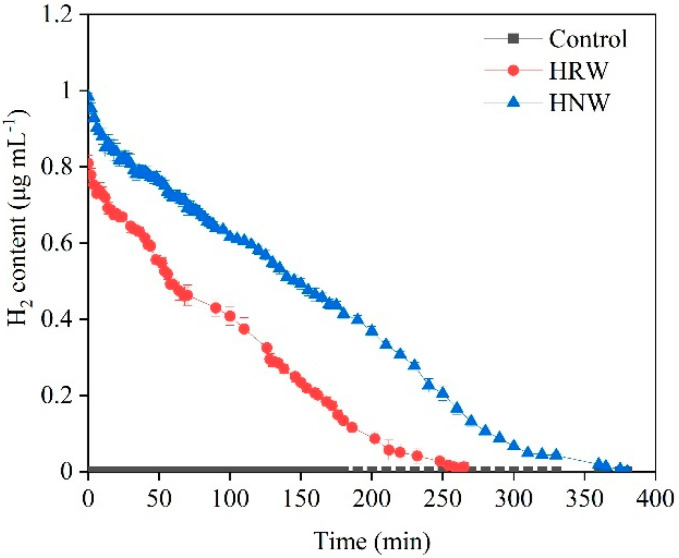
Changes in H_2_ content of fresh hydrogen nanobubble water (HNW) and hydrogen-rich water (HRW).

As shown in Figure 1, the initial content of H_2_ in fresh HNW (about 1.0 μg mL^−1^; regarded as 100% saturation HNW) was higher than that in ordinary fresh HRW without nanobubbles (about 0.8 μg mL^−1^; also regarded as 100% saturation HRW). Meanwhile, H_2_ remained in HNW for about 6 h, which was longer than that in HRW (about 4 h) as the result of the slower evolution. Afterwards, 100% HNW or 100% HRW was immediately diluted to the required concentration (1%, 5%, 10%, 50%, or 10% (*v/v*); equivalently as about 0.01, 0.05, 0.1, 0.5, or 0.08 μg H_2_ mL^−1^), respectively.

**Figure 2:**
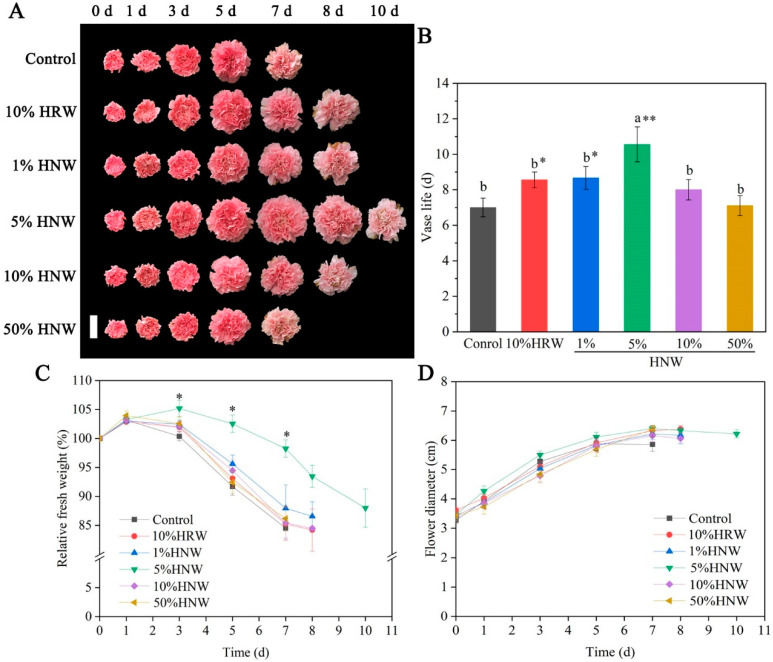
Effects of different concentrations hydrogen nanobubble water (HNW) and 10% hydrogen-rich water (HRW) on morphological changes (**A**), vase life (**B**), relative fresh weight (RFW; (**C**)), and flower diameter (**D**) of cut carnation flowers. Cut flowers were incubated in distilled water (control), 1%, 5%, 10%, and 50% HNW, and 10% HRW for 3 d (changed daily), and then, in distilled water, which was replaced daily until the end of experiments. Representative photographs of cut flowers were taken (scale bar = 4 cm). Afterward, vase life (**B**), RFW (**C**), and flower diameter (**D**) were expressed as mean ± standard error (SE). There were three replicates and three flowers per each. Experiments were conducted three times. The different letters in a column indicated significant differences according to Duncan’s multiple range test (*p* < 0.05), and asterisks indicated significant differences in comparison with the control at *p* < 0.05 (*) or 0.01 (**) according to *t*-test.

Cut carnation flowers incubated in distilled water (control) and HNW/HRW with different concentrations were photographed to document the symptoms of senescence. Compared with distilled water, 1%, 5%, 10% HNW, or 10% HRW differentially delayed the petal wilting and flower withering (Figure 2A), while no significant response was observed for 50% HNW. The treatments with 1%, 5% HNW, and 10% HRW significantly prolonged the vase life of cut carnation flowers, which were assessed as 8.7 ± 0.7 d, 10.6 ± 1.0 d, or 8.6 ± 0.4 d, that prolonged vase life by 23.9%, 50.9%, or 22.3%, respectively, over the H_2_-free control (7.0 ± 0.5 d; Figure 2B). Notably, 5% HNW displayed the most obvious positive effect, which was also greater than 10% HRW. 

During vase life period, the relative fresh weight (RFW) of cut carnation flowers initially increased and then decreased (Figure 2C). Five percent HNW remarkably extended the RFW increase for 3 days, then postponed and slowed down the weight loss on the following days, in comparison with other treatments. However, compared with the distilled water, HNW or HRW had no significant effect on flower diameters (Figure 2D). Since 5% HNW treatment displayed the optimal vase life response, it was used for the subsequent experiments.

### 2.2. Effects of HNW on Relative Water Content and Electrolyte Leakage of Cut Carnation Flowers 

**Figure 3:**
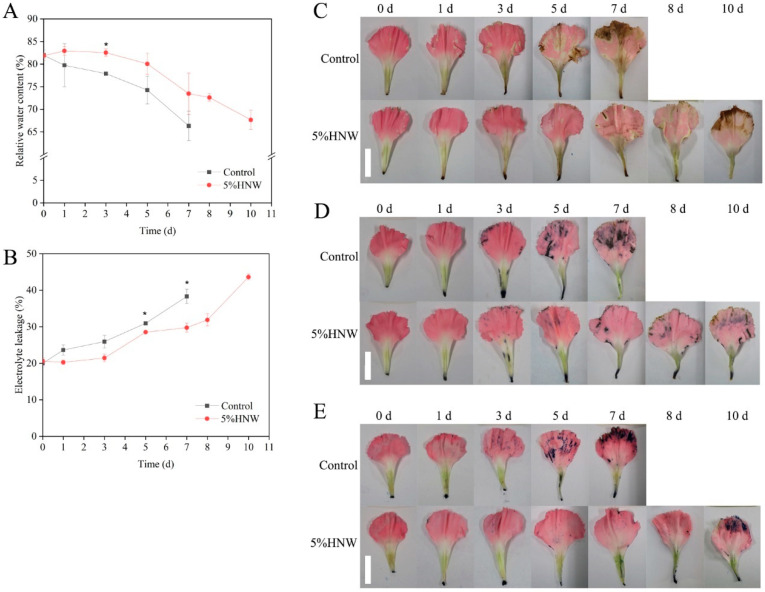
Changes in relative water content (RWC; (**A**)), electrolyte leakage (**B**), hydrogen peroxide (H_2_O_2_; (**C**)), and superoxide anions (O_2_^−^; (**D**)) accumulation as well as cell death (**E**) of carnation petals during vase period. Cut flowers were incubated in distilled water (control) and 5% HNW, respectively, for 3 d (changed daily), and then, in distilled water, which was replaced daily until the end of experiments. Values were expressed as the means ± SE. Asterisks indicated significant differences at *p* < 0.05 by *t*-test. The petals were stained with 3,3-diaminobenzidine (DAB; (**C**)), nitro blue tetrazolium (NBT; (**D**)), and trypan blue (**E**), respectively, then photographed with digital camera. Scale bar = 4 cm. Three flowers per replicate were selected, and total flowers in triplicate were 9 (3 × 3) for each treatment at each time point, then photographed.

In this experiment, the relative water content (RWC) of cut carnation flowers treated with distilled water was continuously decreased during senescence period (Figure 3A). Comparatively, RWC of carnation petals treated with 5% HNW was significantly higher than that of the control on day 3, while thereafter, it was not obviously different from control.

The changes in electrolyte leakage in petals are supposed to indicate changes in membrane permeability [31]. Electrolyte leakage values of carnation petals increased continuously during senescence period (Figure 3B), confirming that the integrity of cell membrane was gradually impaired. While, 5% HNW postponed the increase in electrolyte leakage and maintained it in lower levels at 5–7 d of vase life, in comparison with the control.

### 2.3. Effects of HNW on Reducing the Oxidative Damage and Cell Death in Carnation Petals 

3,3-Diaminobenzidine (DAB) and nitro blue tetrazolium (NBT) staining was usually used to detect the accumulation of ROS (hydrogen peroxide (H_2_O_2_) and superoxide anions (O_2_^−^). A gradual increase in DAB- and NBT-dependent staining in the control during vase period was observed, respectively (Figure 3C,D), suggesting continuous accumulation of ROS and disrupted cellular redox homeostasis. Comparatively, petals from flowers treated with 5% HNW had a slight staining.

In addition, senescence can cause cells death, which was commonly detected by trypan blue staining. As expected, results of trypan blue staining showed that petals treated with 5% HNW exhibited slight blue coloration (Figure 3E). These staining results indicated that the accumulation of ROS and cell death of petals were delayed by 5% HNW.

### 2.4. Effects of HNW on the Activities of DNase, RNase, and Protease

**Figure 4:**
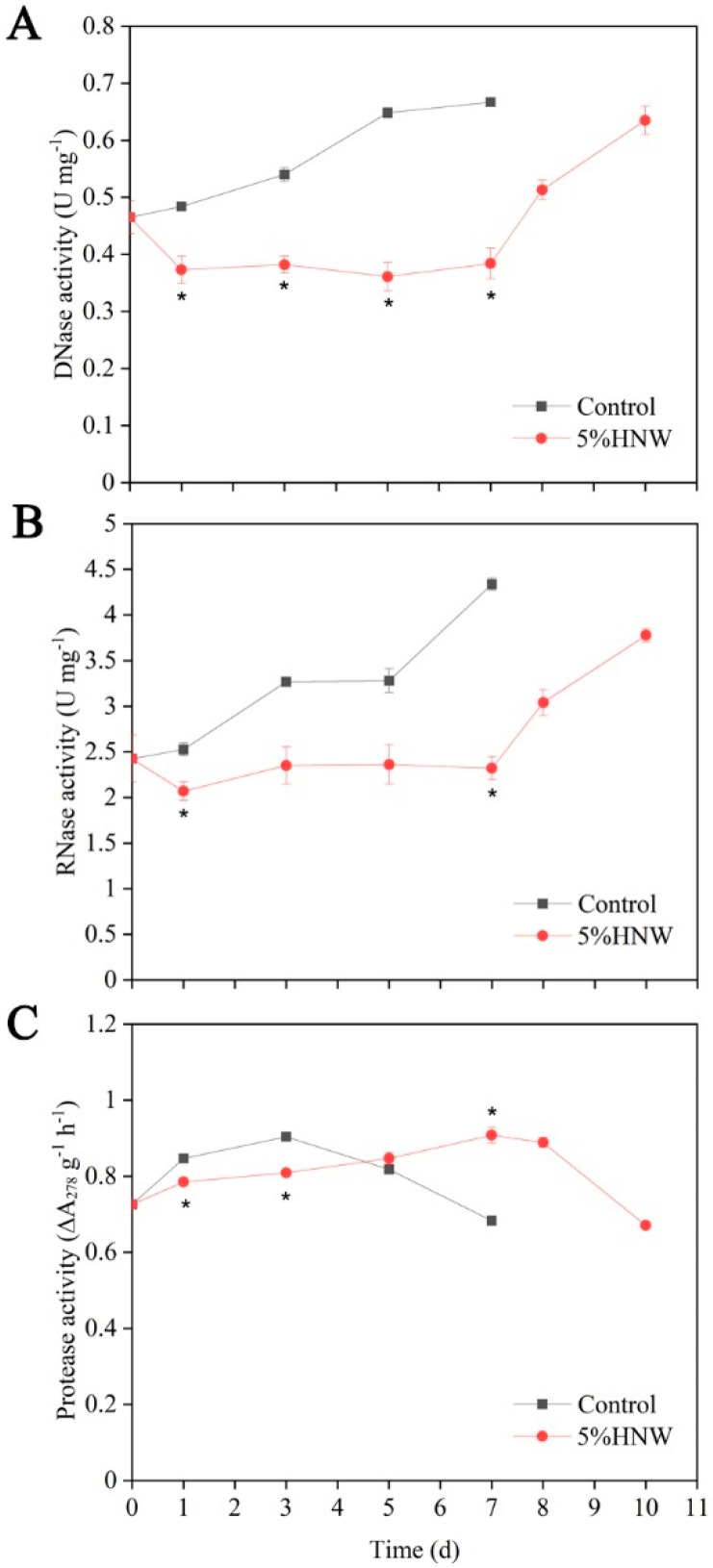
Effects of hydrogen nanobubble water (HNW) on the activities of DNase (**A**), RNase (**B**), and protease (**C**) in petals. Cut flowers were incubated in distilled water (control) and 5% HNW for 3 d (changed daily), and then, in distilled water, which was replaced daily until the end of experiments. Values were expressed as the means ± SE. Three samples per replicate were selected, and total samples in triplicate were 9 (3 × 3) for each treatment at each time point. Asterisks indicated significant differences at *p* < 0.05 according to *t*-test.

To further investigate the contribution of HNW, changes in the enzymatic activities of nucleases (including DNase and RNase) and protease were determined. These assays showed that the activities of DNase and RNase gradually increased during senescence in the control sample (Figure 4A,B). By contrast, when treated with 5% HNW, the activities of DNase and RNase initially decreased and remained at a low level up to day 7, then increased during the last 3 days. Additionally, protease activity in the control petals initially increased (3 d) and, thereafter, declined to some extent (Figure 4C). Compared with the control, the peak of protease activity was postponed to the 7th day by 5% HNW treatment.

## 3. Discussion

It has been found that HRW can prolong the vase life of cut flowers, such as rose [21], lily [22], *Lisianthus* [23], and carnation [24]. Consistently, the experiments showed that 10% HRW also prolonged the vase life of cut carnation “Pink Diamond” flowers (Figure 2A). However, the residence time of H_2_ in HRW was commonly shorter, with its half-life in water being about 100 min (Figure 1). Although MgH_2_ as a soil H_2_-storage material was suggested to be an alternative source for H_2_ delivery due to the improvement of the solubility and residence time of dissolved H_2_ [25], MgH_2_ alone could prolong vase life by less than 30%, which was similar with HRW [26]. However, excess magnesium can cause symptoms resembling those of calcium deficiency and decrease the growth of rice and *Echinochloa* [32]. Similarly, in animals, excess magnesium is detrimental to the skeletal growth and development [33]. Therefore, there should be environmental and health concerns regarding magnesium when MgH_2_ is widely used.

Since nanobubbles have unique properties with high internal pressure and negatively charged surface, these can improve the solubility and residence time of gases in liquid [28]. Nanobubbles have been used in water treatment [28] and soil remediation [34], and they also can promote the growth of animals, plants, and microbes [35,36,37]. Although several studies reported that HNW might optimize the composition of gut microbiota in mice [38] and decrease copper toxicity to *Daphnia magna* [30], the effect of HNW on postharvest preservation of cut flowers has not been reported.

As expected, in this study, HNW produced by a hydrogen nanobubble aerator also extended the residence time of H_2_, and its half-life was about 150 min, in comparison with conventional HRW (about 100 min; Figure 1).

Among different doses of HNW, 5% HNW had the optimal effect on prolonging the vase life of cut carnation flowers, even better than 10% HRW (Figure 2A). Particularly, 5% HNW prolonged vase life by 51%, which was also larger than the effect of MgH_2_ alone (prolonged vase life by 27% [25] and 29% [26]), even close to the combination of MgH_2_ and citrate buffer solution (prolonged vase life by 52% [25]). Besides, HNW may be a good solvent for other additives to achieve a better preservation effect due to its simple composition.

Similar to the previous studies using higher content of HRW [21,23], 50% HNW cannot delay flower senescence, which may be attributable to its hypoxic effects [13,39], confirming that the effect of HNW on cut flower senescence was dose dependent in a specific range. Correlating with the changes in vase life, fresh weight, and flower diameter (Figure 2B–D), as well as the above H_2_ content and residence time (Figure 1), it was suggested that 5% HNW significantly improved the availability of H_2_ and prolonged the vase life of the flowers.

More importantly, the application of HNW is accordingly considered as a superior H_2_ delivery method, and molecular hydrogen is completely harmless to environment and also beneficial to human health [40]. With the increasing home use of H_2_ generator, the consumption cost of H_2_ is reducing to as low as about 10 ¢/mg (https://h2hubb.com/2020/12/08/what-are-the-best-hydrogen-water-generators/, last accessed on 12 August 2021), about 10 ¢/1 L HNW.

Therefore, HNW may have wide-spread application, not only improving people’s health, but also keeping cut flower fresh, thus making life beautiful.

Since water deficit results in flower wilting, maintaining cell turgor could delay cut flower senescence and improve their vase life [1]. It has been demonstrated that H_2_ could enhance water conservation of alfalfa seedling leaves under drought stress [41] and rice root upon boron stress [42], as well as maintain a high level of RWC in cut lily and rose flowers during the vase period [21]. We previously observed that MgH_2_- and HRW-supplied H_2_ could delay RWC reduction in cut carnation flowers [26]. In the present study, HNW had a similar effect to RWC, resulting in alleviation of carnation petal wilting (Figure 2A and Figure 3A).

ROS levels rise during flower senescence, which are highly detrimental for the protein stability and membrane integrity, thus contributing to the cell death and hastening flower senescence [43]. In this study, HNW reduced ROS accumulation induced by senescence, thus maintaining the membrane integrity (Figure 3B–D). These results were consistent with previous studies of the effects of ordinary HRW on cut rose, lily, and *Lisianthus* flowers [21,23]. The enhanced antioxidant capacity by H_2_ was previously proposed as a primary mechanism in plant response against different stresses [12,13,27] and postharvest preservation of fruits [18] and flowers [21,23]. In a previous study, it was confirmed that at similar H_2_ contents, HNW showed higher antioxidant activity than HRW without nanobubbles [29].

Both nucleic acid and protein degradation, resulted from the increased activities of nucleases and protease, play important roles in flower senescence [43]. In this study, the trends towards increased activities of DNase, RNase, and protease were observed during the vase life of cut carnation flowers (Figure 4). Interestingly, HNW decreased or delayed the activities of above three enzymes. Combined with data of cell death (Figure 3E) and phenotypes (Figure 2), we further propose that initial inhibition of the activities of nucleases and protease induced by HNW may partially contribute to alleviate cell death, thus delaying senescence and prolonging the vase life of cut flowers.

In conclusion, the present results clearly showed that, compared with HRW, the supply of HNW-mediated H_2_ increased availability of H_2_, which has a greater potential for application in horticulture. Furthermore, they also demonstrated remarkable roles of HNW in prolonging the vase life of cut flowers by reducing ROS accumulation and inhibiting the activities of nucleases and protease. These findings are expected to open a new window for low-carbon agriculture since H_2_ has unique properties of renewable and zero greenhouse gas emissions on combustion.

## 4. Materials and Methods

### 4.1. Preparation of Hydrogen Nanobubble Water and Hydrogen-Rich Water

HNW was produced by a hydrogen nanobubble water generator (HIM-22; Guangdong Cawolo Health Technology Co., Ltd., Foshan, Guangdong, China). H_2_ produced from water electrolysis was infused into 500 mL distilled water by a nanobubble aerator for 30 min. Conventional HRW was obtained by a H_2_ generator (SHC-300; Saikesaisi Hydrogen Energy Co., Ltd., Jinan, Shandong, China), according to the previous method [23]. H_2_ was bubbled into 500 mL distilled water at a rate of 150 mL min^−1^ for 30 min. The freshly prepared HNW/HRW (1 mg mL^−1^ and pH 8.6 ± 0.4/0.8 mg L^−1^ and 8.4 ± 0.3) was defined as 100% saturation HNW/HRW. Afterwards, 100% HNW or 100% HRW was immediately diluted to required concentration (1%, 5%, 10%, 50%, or 10% (*v/v*)), respectively. The concentrations of dissolved H_2_ were measured by a portable dissolved hydrogen meter (CT-8023; Shenzhen Kedida Electronics Co., Ltd., Shenzhen, Guangdong, China; calibrated by gas chromatography). The mean diameter of H_2_ nanobubbles in HNW was about 300 nm (determined by the NS300, Malvern Panalytical, Britain).

### 4.2. Plant Material and Treatments

Fresh cut carnation “Pink Diamond” flowers (within 1 d after harvest) were purchased from Hanzhongmen Flower Market (Nanjing, Jiangsu Province, China) and immediately transferred to the laboratory within 1 h. The cut carnations with the same degree of openness (the petals elongated vertically) and no mechanical damage were selected and placed in distilled water for 4 h. Afterwards, the stems were cut to a length of 25 cm under the water and the uppermost two leaves were kept.

Subsequently, cut carnations were incubated in distilled water (control), 1%, 5%, 10%, and 50% HNW and 10% HRW for 3 d (changed daily), and then, in distilled water, which was replaced daily until the end of experiments. Since 5% HNW showed the most obvious effect; it was used for the subsequent physiological and biochemical experiments. During the vase period, cut carnations were placed in an incubator at 25 °C and 80–85% relative humidity under a 12 h light/12 h dark photoperiod.

### 4.3. Measurement of Vase Life, Fresh Weight, and Flower Diameter

The vase life of cut carnation flower was calculated as the number of days from the day when flowers were placed in the vase solution until the day that 50% flowers wilted or had bent-neck (bent-neck angle greater than 45°). The fresh weight of cut flowers was measured daily, and relative fresh weight (RFW) was calculated as everyday fresh weight of cut flower against the initial day (0 d). Besides, flower diameter was determined as the maximum diameter of each flower and measured by using a caliper. There were three replicates and three flowers per each. Experiments were conducted in triplicate.

### 4.4. Determination of Water Content and Electrolyte Leakage of Petals

Relative water content (RWC) and electrolyte leakage of petals were measured according to the previous methods [21]. The fresh petals were weighed (*W_f_*) and immersed in distilled water for 6 h at room temperature. Then, the turgid petals were dried and weighed (*W_e_*). The turgid petals were oven dried at 80 °C to a constant weight and weighed again (*W_d_*). RWC was calculated by the formula: RWC (%) = [(*W_f_* − *W_d_*)/(*W_e_* − *W_d_*)] × 100.

Petals (0.2 g) were punched into 1 cm (diameter) discs and immersed in tubes with 20 mL distilled water for 4 h at room temperature after vacuuming for 30 min. Then, the initial conductivity (*C*_0_) was determined. After incubating in boiling water for 15 min and cooling to room temperature, the conductivity (*C*) was determined again. The electrolyte leakage was calculated by the formula: electrolyte leakage (%) = (*C*_0_/*C*) × 100.

For determination of above two parameters, three flowers per replicate were selected, and total flowers in triplicate were 9 (3 × 3) for each treatment at each time point.

### 4.5. Histochemical Staining

ROS (H_2_O_2_ and O_2_) accumulation was detected by DAB and NBT staining, respectively, according to the methods described previously [25,44]. The carnation petals were incubated in 0.1% (*w/v*) DAB or 0.1% (*w/v*) NBT solution for 12 h or 2 h in the dark at room temperature, respectively.

Trypan blue can only penetrate the membranes of dead cells, resulting in staining. The status of cell death in petals was detected by trypan blue staining according to previous method with minor modifications [45]. The petals were incubated in 2.5 mg mL^−1^ trypan blue solution for 1 h at room temperature. After washing extensively, the petals were photographed. Staining was performed with three flowers per replicate, and total flowers in triplicate were 9 (3 × 3) for each treatment at each time point.

### 4.6. Assays of Enzymatic Activity

Samples of 0.5 g fresh petals were homogenized in 3 mL of 0.1 M precooled acetic acid–sodium acetate buffer (pH 5.5) containing 1.1% polyvinylpyridoxone (PVP) in ice bath. The mixture was centrifuged at 12,000 rpm at 4 °C for 10 min. The supernatant was used for DNase and RNase activity assay according to the colorimetric method described previously [46]. For the determination of DNase activity, 200 μL of enzyme extract with 200 μL denatured calf thymus DNA (1 mg mL^−^^1^) were incubated in 37 °C water bath for 1 h. Afterwards, the reaction was terminated by adding 95% (*v/v*) ethyl alcohol and settled at −20 °C for 12 h. The mixture was centrifuged at 12,000 rpm at 4 °C for 10 min. The absorbance of the supernatant was measured at 260 nm with a blank incubation without enzyme. One unit of enzyme activity was defined as the amount of enzyme causing an increased absorbance of 1.0 in 1 h at 260 nm. RNase activity was determined similarly with the following modification: yeast RNA (10 mg mL^−^^1^) was used instead of calf thymus DNA (1 mg mL^−^^1^). Enzyme activity was expressed as U mg^−1^ protein. Protein content was determined according to the Bradford method with BSA as a standard [47].

According to previous method [48], protease activity was determined by using hemoglobin as the substrate. The reaction mixture (0.2 mL 2% (*w/v*) denatured hemoglobin solution, 0.2 mL acetic acid–sodium acetate buffer (0.1 M, pH 5.2), and 0.2 mL crude extract) was incubated in 37 °C water bath for 1 h. The reaction was terminated by the addition of 0.8 mL 7.5% (*w/w*) trichloroacetic acid (TCA) and settled at 4 °C for 30 h. After centrifugation, the absorbance of the supernatant was measured at 278 nm against control that was added with TCA before reaction. Enzyme activity was expressed as ∆A_278_ g^−1^ (fresh weight) h^−1^.

For activity assays of above three enzymes, three samples per replicate were selected, and total samples in triplicate were 9 (3 × 3) for each treatment at each time point.

### 4.7. Statistical Analysis

All values are expressed as the mean ± SE from three independent experiments for each treatment. Statistical analysis was performed by using SPSS 22.0 software (IBM Corporation, Armonk, NY, USA). The significant difference among treatments were analyzed by Duncan’s multiple range test or *t*-test (*p* < 0.05 or 0.01).

## Data Availability

All data, models, and code generated or used during the study appear in the submitted article.

## Abbreviations

DAB	3,3-diaminobenzidine
RFW	relative fresh weight
H_2_	hydrogen gas
HNW	hydrogen nanobubble water
HRW	hydrogen-rich water
H_2_O_2_	hydrogen peroxide
NBT	nitro blue tetrazolium
O_2_^−^	superoxide anions
PVP	polyvinylpyridoxone
RFW	relative fresh weight
ROS	reactive oxygen species
RWC	relative water content
SE	standard error
